# Hematologic System Dysregulation in Critically Ill Septic Patients with Anemia—A Retrospective Cohort Study

**DOI:** 10.3390/ijerph19116626

**Published:** 2022-05-29

**Authors:** Piotr F. Czempik, Jan Herzyk, Dawid Wilczek, Łukasz J. Krzych

**Affiliations:** 1 Department of Anaesthesiology and Intensive Care, Faculty of Medical Sciences in Katowice, Medical University of Silesia, 40-752 Katowice, Poland; lkrzych@sum.edu.pl; 2 Students’ Scientific Society, Department of Anaesthesiology and Intensive Care, Faculty of Medical Sciences in Katowice, Medical University of Silesia, 40-752 Katowice, Poland; s76420@365.sum.edu.pl (J.H.); s76509@365.sum.edu.pl (D.W.)

**Keywords:** anemia, coagulopathy, critical illness, outcome, red blood cell distribution width, sepsis, septic shock

## Abstract

Sepsis can affect various organs as well as the hematologic system. Systemic dysregulation, present in sepsis, affects particularly red blood cells (RBCs). One of the widely available RBC indices is RBC distribution width (RDW). Sepsis may also affect hemostasis, with septic patients presenting with coagulopathy or disseminated intravascular coagulation. The aim of our study was to analyze the impact of sepsis on RBC indices and coagulation parameters on admission to the intensive care unit (ICU) and their association with presence of sepsis and sepsis outcomes in anemic critically ill patients. We performed a retrospective observational study covering consecutive patients admitted to a 10-bed mixed ICU in the years 2020–2021. We found significant differences between septic and non-septic patients for the following parameters: RDW (*p* = 0.02), INR (*p* < 0.01), aPTT (*p* < 0.01), D-dimers (*p* < 0.01), fibrinogen (*p* = 0.02), platelets (*p* = 0.04). International normalized ratio was the only parameter with adequate sepsis predictive value (AUROC = 0.70; 95% CI 0.63–0.76; *p* < 0.01), with an optimal cut-off value of >1.21. Combination of INR with fibrinogen and a severity of disease score improved INR’s predictive value (AUROC 0.74–0.77). Combination of INR with a severity of disease score was an adequate ICU mortality predictor in septic patients (AUROC 0.70–0.75). Sepsis significantly affects RDW and most coagulation parameters. Increased INR can be used for sepsis screening, whereas combination of INR with a severity of disease score can be a predictor of short-term mortality in septic patients.

## 1. Introduction

Sepsis and septic shock are frequent diagnoses on admission to the intensive care unit (ICU). Incidence of ICU-treated sepsis has been recently estimated at 58 per 100,000 person-years [1]. Moreover, the most recent Surviving Sepsis Campaign guidelines suggest that patients with sepsis or septic shock should be admitted to the ICU within 6 h of sepsis diagnosis [2].

According to the most recent consensus-based international definition published in 2016, sepsis is “a life-threatening organ dysfunction caused by a dysregulated host response to infection” [3]. Previous definitions assumed inappropriate continuity of severity of disease from severe sepsis to septic shock. Moreover, systemic inflammatory response syndrome (SIRS) criteria lacked appropriate sensitivity and specificity. Septic shock has recently been defined as a specific subtype of sepsis in which circulatory, cellular, and metabolic disturbances lead to increased mortality [3]. Diagnostic criteria for septic shock comprise hypotension not responding to initial intravenous fluid resuscitation, hence there is a need for commencing vasopressor (i.e., norepinephrine), and metabolic disturbances which are reflected by increased blood lactate. Mortality in sepsis and septic shock generally varies between one in six to one in three patients [4]. Hospital mortality of ICU-treated septic patients could be as high as nearly 42% [1]. Sepsis is a medical emergency condition in which survival decreases with each hour of delay in commencing appropriate treatment [5].

Every effort should be made to establish diagnosis of sepsis or septic shock in a timely fashion, therefore healthcare institutions should have sepsis screening programs in place. There are various sepsis screening tools available, characterized by different performance powers calculated as the area under receiving operator characteristic (AUROC) curve: Modified Early Warning Score (MEWS)—0.5; SIRS criteria—0.70; Sequential Organ Failure Assessment (SOFA)—0.78 [6]. In order to timely identify septic patients outside ICU, in an emergency room or a non-high dependency unit hospital ward, clinical score termed quick SOFA (qSOFA) was proposed, in which sepsis is suspected by scoring 2 out of points [3]. The quest for a highly reliable sepsis screening test is ongoing.

Sepsis can affect hematologic system components: RBCs, white blood cells, platelets (PLTs), and coagulation. Systemic dysregulation, which is the principal feature of sepsis, is especially visible in cells involved in oxygen delivery, specifically RBCs [7,8]. Septic dysregulation includes both quantitative and qualitative changes in RBCs. Qualitative alternations in RBCs include changes in: volume, morphology, deformability, aggregation, metabolism, antioxidant status, homeostasis of intracellular ionized calcium [9]. The parameter that reflects changes in RBC volume is RBC distribution width (RDW). RDW is defined as a coefficient of variation of RBC volume (i.e., mean cell volume, MCV) and represents a degree of RBC size heterogeneity [10]. Common, early impact of sepsis on RBC volume, and wide availability of RDW in clinical practice, means that it can be used as a screening parameter or an outcome predictor in sepsis. The role of RDW in prediction of mortality in sepsis was extensively researched, however studies showed conflicting results. Whereas some researchers linked RDW with increased morality in sepsis [11] and septic shock [11,12], others questioned this correlation [12,13]. Other qualitative changes in RBCs—alterations of morphology and deformability—have been found to be early indicators of sepsis [14]. Aforementioned quantitative and qualitative RBC changes lead to anemia. Anemia associated with sepsis is categorized as anemia of inflammation. This type of anemia is difficult to diagnose using standard laboratory tests [15]. Septic patients may also present with pre-existing iron-deficiency. Standard iron status tests are deranged by systemic inflammatory response, therefore iron-deficiency anemia is also difficult to diagnose in septic patients [16].

Abnormal values for standard laboratory tests of coagulation are often reported in critically ill patients. Widely available standard laboratory tests of coagulation include: activated partial thromboplastin time (aPTT), prothrombin time (PT), thrombin time (TT), fibrinogen concentration (Clauss method), and platelet count (PLT). Septic patients may present with sepsis-associated coagulopathy with prolonged PT and low PLT, as well as disseminated intravascular coagulation (DIC), characterized by prolonged PT, low PLT, low fibrinogen, and elevated fibrin-related marker (e.g., D-dimers) [17]. Sepsis causes incremental derangement of these widely available standard laboratory tests of coagulation. This effect could be used for screening and outcome prediction in sepsis.

The primary aim of our study was to assess the impact of sepsis on complete blood count (CBC) RBC indices, PLT, and standard laboratory tests of coagulation in anemic patients on admission to ICU. The secondary aim of our study was to analyze associations of CBC RBC indices, PLT and standard laboratory tests of coagulation with presence of sepsis/septic shock, short-term mortality (ICU mortality), severity of sepsis (according to SOFA), ICU length of stay (ICU-LOS).

## 2. Materials and Methods

**Figure 1:**
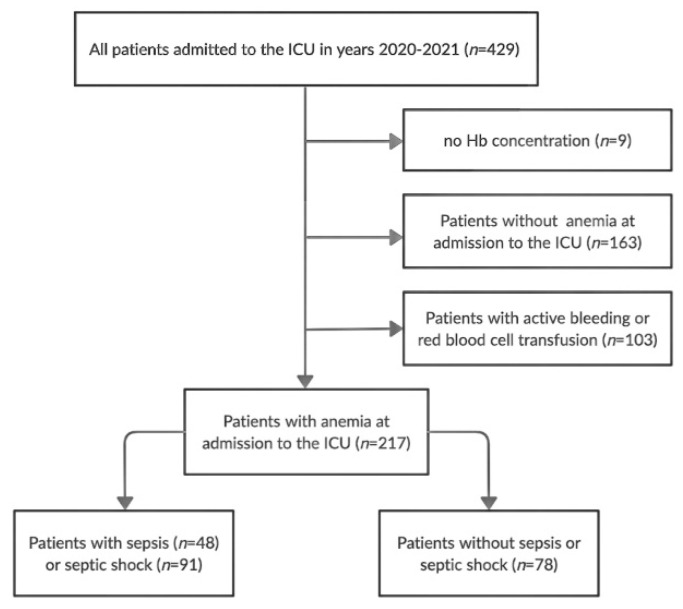
The study flow chart. Hb—hemoglobin, ICU—intensive care unit.

We performed a retrospective observational study covering anemic patients admitted to a 10-bed mixed surgical-medical ICU in a tertiary care teaching hospital in the years 2020–2021. We excluded patients with active medical/surgical bleeding or in whom RBC was transfused (*n* = 103), patients without anemia (*n* = 163), and patients in whom hemoglobin (Hb) was not determined due to short ICU-LOS caused by rapid death (*n* = 9). The study flow chart is presented in Figure 1.

Demographic, clinical, and laboratory data were retrieved from the electronic health records (AMMS, Asseco, Rzeszow, Poland). The severity of illness on admission to ICU was assessed using three acknowledged classification systems: Simplified Acute Physiology Score II (SAPS II), Acute Physiology and Chronic Health Evaluation (APACHE II), and Sepsis-related Organ Failure Assessment (SOFA). For diagnosis of sepsis and septic shock we used recent sepsis-3 and septic shock-3 definitions, respectively [3]. Anemia was defined as Hb concentration below the local laboratory reference value, determined in a blood sample collected in a standard ethylenediaminetetraacetic acid (EDTA) test tube (BD Vacutainer K2EDTA 2 mL, Beckton Dickinson, Dublin, Ireland), collected to perform CBC with differentiation. We analyzed CBC RBC indices and PLT in septic and non-septic patients. Complete blood count was performed using an automated hematology analyzer (Sysmex, Warszawa, Poland). Analyzed standard laboratory tests of coagulation included: fibrinogen (Claus method), PT, INR, aPTT, D-dimers, TT. The local laboratory reference ranges for these parameters are presented in Table 1.

The following clinical data were retrieved: source of sepsis (i.e., pulmonary, urinary tract, intraabdominal, bloodstream infection associated with a central line, central nervous system, unspecified), presence of organ injury (i.e., sepsis-associated brain dysfunction, acute respiratory failure, acute kidney injury, acute liver injury, shock). We also retrieved the following laboratory test results: lactate, procalcitonin (PCT), C-reactive protein (CRP), creatinine, blood urea nitrogen (BUN), and bilirubin. The lactate concentration was determined in blood collected in an arterial blood gas (ABG) test tube (BD A-Line™ 1 mL, Beckton Dickinson, Franklin Lakes, NJ, USA). Arterial blood gas analysis was performed using the local blood gas analyzer (RAPIDPoint^®^ 500, Siemens Healthcare, Erlangen, Germany). All other laboratory tests were determined in blood collected in a standard sodium citrate test tube (BD Vacutainer, Beckton Dickinson, Warsaw, Poland). The primary outcome measure was all-cause short-term mortality (ICU mortality). The secondary outcome measures were severity of disease (according to SOFA score) and ICU-LOS (in days).

Due to retrospective observational character of the study, the local ethics committee waived the requirement for informed consent (PCN/0022/KB/258/19).

Statistical analysis was performed using licensed MedCalc Statistical Software v. 18.0 (MedCalc Software bvba, Ostend, Belgium). Continuous variables were presented as medians and interquartile ranges (IQR). Categorical variables were presented as absolute values and percent. Between-group differences for continuous variables were assessed with Student’s *t*-test or Mann–Whitney U-test. Between-group differences for categorical variables were assessed using chi-squared or Fischer’s exact test. Associations between variables were tested using Spearman rank correlation analysis. The receiver operating characteristic (ROC) curve was drawn and the area under the ROC curve (AUROC) was calculated to analyze the role of investigated parameters as predictors of sepsis and its outcomes. Logistic regression was used to assess the predictive value of different combinations of variables, odds ratios (OR), and their 95% confidence intervals (CI) were calculated. A “*p*” value of <0.05 was considered statistically significant.

## 3. Results

The study population characteristics are presented in Table 2.

Septic vs. non-septic patients scored higher on all severity of disease classification systems and had higher incidence of acute kidney injury, acute liver injury, and shock. Concentrations of lactate, PCT, CRP, creatinine, BUN, and bilirubin were significantly higher in septic vs. non-septic patients. The following sources of sepsis were present in the study population: intraabdominal—60 (43.2%), pulmonary—43 (30.9%), urinary tract—20 (14.4%), central nervous system—7 (5.0%), central line-associated bloodstream infection—1 (0.7%), unspecified—8 (5.8%).

Red blood cell indices and standard laboratory tests of coagulation on admission to ICU are presented in Table 3. Septic patients compared to non-septic patients had significantly higher RDW and RDW-SD, as well as PT, INR, aPTT, D-dimers, fibrinogen. Number of platelets was lower in septic vs. non-septic patients. There were no inter-group differences in RBC indices as far as origin of sepsis or presence of shock was concerned.

**Figure 2:**
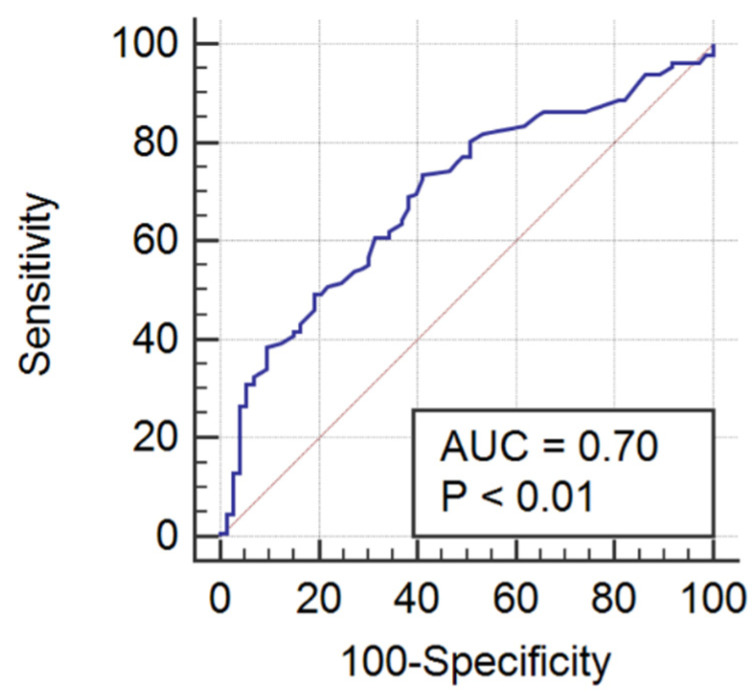
The receiving operating characteristic curve of INR in prediction of sepsis on admission to ICU. ICU—intensive care unit, INR—international normalized ratio.

The AUROC for RDW and RDW-SD in prediction of sepsis on admission to ICU was 0.60 (95% CI 0.53–0.66; *p* = 0.02), with an optimal cut-off value > 15.1% and > 45.1 fL, respectively. Combination of RDW and RDW-SD with a disease severity score did not significantly improve its sepsis predictive power: SOFA (AUROC = 0.68, 95% CI 0.61–0.74), APACHE II (AUROC 0.65, 95% CI 0.58–0.71), SAPS II (AUROC = 0.66, 95% CI 0.59–0.72). International normalized ratio was the only coagulation test with acceptable sepsis prediction power. The ROC curve of INR in prediction of sepsis is shown in Figure 2. The AUROC for INR in prediction of sepsis on admission to ICU was 0.70 (95% CI 0.63–0.76; *p* < 0.01), with an optimal cut-off value of >1.21 (Figure 2).

Combination of INR (OR 7.23, 95% CI 2.15–24.32), and fibrinogen (OR 1.00, 95% CI 1.00–1.00) improved sepsis predictive value (AUROC 0.74, 95% CI 0.66–0.80). Combination of INR and fibrinogen with severity of disease score slightly improved this predictive value: SOFA (AUROC = 0.76, 95% CI 0.68–0.82), APACHE II (AUROC = 0.75; 95% CI 0.68–0.82), SAPS II (AUROC = 0.77; 95% CI 0.69–0.83).

Red blood cell indices and standard laboratory tests of coagulation in survivors and non-survivors with sepsis or septic shock are presented in Table 4. We found significant differences in PT and INR between survivors and deceased patients with sepsis or septic shock, with deceased patients having prolonged PT and increased INR.

The AUROC for INR in predicting ICU mortality in septic patients was 0.67 (95% CI 0.59–0.73; *p* < 0.01), with an optimal cut-off value of >1.47. Combination of INR with a disease severity score improved its predictive power: with SOFA (AUROC 0.70, 95% CI 0.62–0.78, *p* < 0.01), APACHE II (AUROC 0.74, 95% CI 0.66–0.81, *p* < 0.01), SAPS II (AUROC = 0.75; 95% CI 0.68–0.82).

We found that non-survivors with sepsis or septic shock scored higher on all three disease severity classification systems: SAPS II [56.0 (IQR 43.2–66.7) vs. 39.0 (IQR 30.5–56.0); *p* < 0.01], APACHE II [23.0 (IQR 17.0–29.0) vs. 15.5 (IQR 11.0–23.0); *p* < 0.01], and SOFA [11.0 (IQR 7.0–13.7) vs. 8.0 (IQR 5.0–11.5); *p* < 0.01].

Results of correlation analysis between analyzed parameters, SOFA score and ICU-LOS are presented in Table 5.

There was no correlation between RBC indices and severity of sepsis or ICU-LOS.

We found positive correlations between PT, INR, D-dimers, TT, and severity of sepsis according to SOFA score. There was negative correlation between PLT and SOFA score. There was only one parameter that correlated (negatively) with ICU-LOS, specifically PLT.

## 4. Discussion

In our study we showed that RDW and RDW-SD were significantly higher in septic than non-septic patients with anemia. RDW is the coefficient of variation of RBC volume and represents RBC size heterogeneity [10]. It increases when excess reticulocytes are present in the circulation. Red blood cell distribution width may be increased by destruction of RBCs, nutritional deficiencies, and RBC transfusion. Nutritional deficiencies leading to increased RDW include iron, vitamin B12, and folate [18]. Inflammation and the associated oxidative stress shortens RBC survival and impairs RBC maturation, leading to release of premature RBCs, contributing to elevated RDW [19]. Pro-inflammatory cytokines also inhibit erythropoietin-induced erythrocyte proliferation and maturation, also leading to elevated RDW [20]. Oxidative stress is ubiquitous in inflammatory conditions such as sepsis or septic shock [21]. Increased RDW has been associated with increased B-type natriuretic peptide in patients with coronary artery disease [22]. Increased RDW in our study was most certainly caused by SIRS present in patients with sepsis or septic shock, The degree of SIRS could be judged by concentration of inflammatory markers in our septic population, which were much higher in septic than in non-septic patients (PCT 16 times higher; CRP more than 3 times higher).

We analyzed in our study the potential role of RDW and RDW-SD as sepsis markers. These parameters were poor predictors of sepsis on admission to ICU. As search for accurate screening test for sepsis is ongoing, different laboratory markers and prognostic clinical tools have been researched. One of the tools that has been extensively researched as a potential screening marker for sepsis is qSOFA. The qSOFA comprises three variables and was developed for easy prediction (no need for laboratory work-up) of mortality and prolonged (>3 days) ICU-LOS in patients with sepsis or suspected of having sepsis. The score include neurologic status assessment (Glasgow Coma Scale score < 15), respiratory rate ≥ 22 breaths/min), and hypotension (systolic blood pressure ≤ 100 mm Hg). Quick SOFA is a good sepsis predictor outside the ICU (AUROC = 0.81; 95% CI 0.80–0.82), whereas its predictive power in the ICU drops (AUROC = 0.66; 95% CI 0.64–0.68) [23].

We found that RDW and RDW-SD were not associated with short-term mortality in septic/septic shock patients. However some studies found that RDW can be both static (single result) and dynamic (changes in RDW) mortality prognostic marker. In our study we focused on baseline values and we did not perform trend analysis. Kim et al. found that rise in RDW over initial 72 h of hospitalization was a strong and independent predictor of short-term and long-term (90-day) mortality in septic patients [24]. Elevated RDW has been linked to mortality in different cohorts of patients: outpatients [25], stroke patients [26], peripheral artery disease patients [27], patients referred for coronary angiography [28], older patients [29], ICU patients [30,31], sepsis patients [11], septic shock patients [11,12], and neonatal sepsis patients [31]. In one of the studies, RDW was found to be an independent predictor of in-hospital mortality in septic patients with and without anemia [32].

In our study we found that RDW and RDW-SD were not associated with severity of sepsis as judged by SOFA score. Increased RDW has been associated with the severity of disease in patients with coronary artery disease [33] or pulmonary hypertension [34]. As far as other RBC indices are concerned, we did not find significant differences between septic and non-septic patients. Anemic septic patients may have MCV within normal values, however their shape may change from biconcave discs into spherostomatocytes. This sphericity was found to correlate with RBC sialic acid membrane content, suggesting that altered RBC surface charge is a factor here [7]. Nevertheless, correlation between MCV and severity of sepsis in our study was close to statistical significance (*p* = 0.05). Moreover, we found that RDW was not associated with ICU-LOS.

We found significant differences in standard laboratory tests of coagulation between septic and non-septic patients on admission to ICU. Fibrinogen concentration in septic patients in our study was almost 120 mg/dL higher compared to non-septic patients, and was well above the upper reference limit. Fibrinogen is an acute phase protein and its increased synthesis during sepsis results in high plasma levels [35], therefore increased fibrinogen concentration may be used as a marker of sepsis. On the other hand, decreased fibrinogen can be an early sign of sepsis-induced DIC. In this context fibrinogen can be used as a marker of mortality in sepsis, with fibrinogen concentration < 200 mg/dL predicting higher mortality [36]. D-dimers concentration in our study was also higher in septic than non-septic patients. The development of DIC, in which D-dimer is a predictor, has been associated with severe sepsis [37]. High concentration of D-dimers has been a marker of poorer outcome in patients with sepsis in the ICU [38]. Lynos et al. pointed out that hospital mortality in patients without sepsis-associated coagulopathy was 25.4%, but it grew rapidly to 56.1% in patients with sepsis-associated coagulopathy. Furthermore, duration of hospitalization and ICU care increased progressively with severity of sepsis-associated coagulopathy [39]. International normalized ratio in our study was significantly higher in septic than in non-septic patients. We also found INR to be a predictor of sepsis on admission to ICU. This result is similar to other studies, where INR has been found to be an independent prognostic risk factor for sepsis [40]. We found significant differences in PT and INR between survivors and deceased patients with sepsis or septic shock. Combination of INR with one of disease severity scores was an adequate predictor of ICU mortality in septic patients. Prolonged PT and increased INR in patients with sepsis on admission to ICU reflect stronger anomalies in pathways implicated in the pathogenesis of sepsis [40]. In our study, not only PT, but also aPTT was higher in septic than non-septic patients. Activated partial thromboplastin time in septic patients in our study was outside the reference range, whereas in non-septic patients aPTT stayed within the reference range. Dempfle et al. described aPTT as a marker for rapid and highly specific identification of patients with sepsis [41]. We did not find aPTT to be a predictor of sepsis on admission to ICU. In the ICU population, prolonged aPTT has been found to be a marker of both sepsis and increased mortality in septic patients [42]. In our study we did not find aPTT to be a predictor of mortality in septic patients. We found significant differences in PLT between septic and non-septic patients in our study. Number of platelets has been previously described as a prognostic marker for sepsis [43].

There were some limitations to our study. Firstly, the retrospective character of the study did not allow us to exclude other factors having impact on RBC indices. Moreover, due to retrospective character of the study we did not investigate mineral and vitamin deficiencies, neither measured concentration of interleukin-6. Secondly, it is important to underline that our study was a single center study, performed specifically in patients hospitalized in the ICU, which may have limited the generalizability of the results obtained. Thirdly, the number of subjects is a limitation, which could have prevented us from showing correlations between RBC indices (MCV, RDW-SD) and severity of sepsis. Lastly, we did not perform follow-up of patients, so true mortality associated with sepsis in our subjects could not be established.

## 5. Conclusions

Sepsis significantly affects RDW and most coagulation parameters. Increased INR can be used for sepsis screening, whether combination of INR with severity of disease score can be used as a predictor of short-term mortality in septic patients.

## Figures and Tables

**Table 1:** Local laboratory reference ranges for analyzed parameters.

Parameter	Reference Range [Male/Female]
Hemoglobin [g/dL]	13.5–16.5/11.5–15.0
Hct [%]	40–53/36–46
MCV [fL]	84–98
MCH [pg]	27–31
MCHC [pg]	32–36
RDW [%]	11–16
RDW-SD [fL]	36.3–47.3/38.9–50.0
Fibrinogen [mg/dL]	200.0–393.0
PT [s]	9.4–12.5
INR	0.80–1.20
aPTT [s]	25.4–36.9
D-dimers [ng/mL]	<500.0
TT [s]	10.3–16.6
PLT [10^3^/µL]	130–400

aPTT—activated partial thromboplastin time, Hct—hematocrit, INR—international normalized ratio, MCH—mean cell hemoglobin, MCHC—mean cell hemoglobin concentration, MCV—mean cell volume, PLT—platelets, PT—prothrombin time, RDW—red cell distribution width, RDW-SD—standard deviation of red cell distribution width, TT—thrombin time.

**Table 2:** Study population characteristics.

Characteristic	All Patients	Septic Patients	Non-Septic Patients	*p*
	(*n* = 217)	(*n* = 139)	(*n* = 78)	
Age [years]	65 (58–73)	66 (58–74)	67 (56.5–71.2)	0.57
Sex, female/male [*n*, %]	98(45.2)/	62(44.6)/	36(46.2)/	0.83
	119(54.8)	77(55.4)	42(53.8)	
Severity of disease:				
- SAPS II [points]	44 (32–59)	49 (36–62)	39 (29–49)	<0.01
- APACHE II points]	18 (13–24)	20(14–26)	16 (12–21)	<0.01
- SOFA II [points]	8 (6–12)	10 (6–13)	7 (4–9)	<0.01
Organ injuries:				
- acute respiratory failure [*n*, %]	199 (91.7)	127 (91.4)	72 (92.3)	0.81
- acute kidney injury [*n*, %]	99 (45.6)	86 (61.9)	13 (16.7)	<0.01
- acute liver injury [*n*, %]	46 (21.2)	41 (29.5)	5 (6.4)	<0.01
- shock [*n*, %]	122 (56.2)	91 (65.5)	31 (39.7)	<0.01
Selected laboratory tests:				
- lactate [mmol/L]	2.5 (1.7–4.1)	2.8 (1.9–4.7)	1.9 (1.8–2.3)	<0.01
- PCT [ng/mL]	1.2 (0.33–7.18)	3.2 (1.18–17.0)	0.2 (0.1–0.4)	<0.01
- CRP [mg/L]	127.6 (57.9–228.5)	192 (98.9–266.0)	61.2 (32.5–115.0)	<0.01
- Creatinine [mg/dL]	1.3 (0.8–2.2)	1.5 (0.8–2.7)	0.9 (0.8–1.1)	<0.01
- BUN [mg/dL]	32.3 (19.4–49.6)	38.3 (23.1–57.4)	23.5 (17.1–36.6)	<0.01
- Bilirubin [mg/dL]	0.7 (0.5–1.3)	0.8 (0.5–1.6)	0.6 (0.4–0.8)	<0.01
All-cause ICU mortality [*n*, %]	96 (44.2)	75 (53.9)	20 (25.6)	<0.01

Categorical data are presented as absolute values and percent. Continuous data are presented as medians and interquartile ranges (IQR) (in brackets). APACHE II—Acute Physiology and Chronic Health Evaluation, BUN—blood urea nitrogen, CRP—C-reactive protein, ICU—intensive care unit, IQR—interquartile range, PCT—procalcitonin, SOFA—Sepsis-related Organ Failure Assessment, SAPS II—Simplified Acute Physiology Score II.

**Table 3:** Analyzed parameters on admission to ICU in septic and non-septic patients.

Parameter	All Patients	Septic Patients	Non-Septic Patients	*p*
Hb [g/dL]	10.2 (9.1–11.2)	10.0 (9.1–11.1)	10.5 (9.2–11.3)	0.13
Hct [%]	30.6 (27.8–34.0)	30.2 (27.4–33.8)	31.6 (28.3–35.0)	0.05
MCV [fL]	89.4 (85.7–94.4)	89.4 (86.5–94.6)	89.4 (84.0–94.0)	0.39
MCH [pg]	29.9 (28.4–31.1)	29.9 (28.7–31.1)	29.8 (28.0–31.2)	0.48
MCHC [pg]	33.1 (31.7–34.0)	33.1 (31.7–34.1)	33.1 (31.3–34.0)	0.54
RDW [%]	15.3 (13.9–17.2)	15.7 (14.2–17.2)	14.8 (13.6–16.5)	0.02
RDW-SD [fL]	49.9 (45.9–55.8)	51.0 (46.9–56.9)	48.9 (43.8–53.1)	0.02
Fibrinogen [mg/dL]	480.0 (326.2–647.0)	518.5 (344.5–674.5)	402.0 (280.2–604.0)	0.02
PT [s]	15.0 (13.1–17.8)	15.9 (13.8–18.8)	13.5 (12.6–15.6)	<0.01
INR	1.3 (1.1–1.6)	1.4 (1.2–1.6)	1.2 (1.1–1.4)	<0.01
aPTT [s]	36.7 (30.2–43.1)	37.7 (31.7–44.3)	33.2 (29.8–38.7)	<0.01
D-dimers [mcg/mL]	5175.0 (2265.2–7626.2)	5681.0 (2765.0–12,771.7)	3449.5 (1445.5–6190.5)	<0.01
TT [s]	17.6 (15.9–21.0)	17.8 (15.8–20.4)	17.5 (16.9–21.1)	0.62
PLT [10^3^/µL]	212.0 (140.7–316.5)	195.0 (128.0–315.2)	239.0 (176.0–320.0)	0.04

Values are medians and interquartile ranges (IQR) (in brackets). aPTT—activated partial thromboplastin time, Hb—hemoglobin, Hct—hematocrit, INR—international normalized ratio, MCH—mean cell hemoglobin, MCHC—mean cell hemoglobin concentration, MCV—mean cell volume, PLT—platelets, PT—prothrombin time, RDW—red cell distribution width, RDW-SD—standard deviation of red cell distribution width, TT—thrombin time.

**Table 4:** Red blood cell indices and standard laboratory tests of coagulation in survivors and non-survivors with sepsis or septic shock.

Parameter	Survivors	Non-Survivors	*p*
Hb [g/dL]	10.0 (9.1–11.0)	10.0 (8.7–11.1)	0.95
Hct [%]	30.1 (27.6–33.0)	30.3 (27.2–34.6)	0.62
MCV [fL]	88.4 (85.9–95.6)	91.2 (86.8–94.5)	0.21
MCH [pg]	29.8 (28.9–31.4)	29.9 (28.5–31.1)	0.61
MCHC [pg]	33.3 (32.2–34.2)	32.9 (31.6–33.9)	0.08
RDW [%]	15.3 (14.0–17.4)	15.9 (14.4–17.2)	0.38
RDW-SD [fL]	50.4 (46.1–57.1)	51.3 (47.2–56.6)	0.40
Fibrinogen [mg/dL]	574.5 (335.0–770.0)	505.5 (357.5–635.5)	0.41
PT [s]	14.8 (13.2–16.3)	17.7 (14.5–19.6)	<0.01
INR	1.3 (1.1–1.4)	1.5 (1.3–1.7)	<0.01
aPTT [s]	37.0 (30.0–44.3)	38.4 (32.8–44.6)	0.25
D-dimers [mcg/mL]	5157.0 (2351.0–7321.5)	6513.5 (4199.0–13,339.5)	0.07
TT [s]	16.8 (15.1–19.5)	18.1 (15.9–22.8)	0.09
PLT [10^3^/µL]	203.0 (137.0–344.5)	189.0 (109.0–304.7)	0.39

Values are medians and interquartile ranges (IQR) (in brackets). aPTT—activated partial thromboplastin time, Hb—hemoglobin, Hct—hematocrit, INR—international normalized ratio, MCH—mean cell hemoglobin, MCHC—mean cell hemoglobin concentration, MCV—mean cell volume, PLT—platelets, PT—prothrombin time, RDW—red cell distribution width, RDW-SD—red cell distribution width-standard deviation, TT—thrombin time.

**Table 5:** Associations between analyzed parameters, sepsis severity and intensive care unit length of stay.

Parameter	SOFA Score	*p*	ICU Length of Stay	*p*
Hb [g/dL]	−0.06	0.45	−0.09	0.30
Hct [%]	−0.03	0.69	−0.08	0.36
MCV [fL]	0.17	0.05	0.06	0.94
MCH [pg]	0.07	0.43	−0.06	0.49
MCHC [pg]	−0.13	0.14	−0.03	0.69
RDW [%]	0.06	0.47	−0.03	0.69
RDW-SD [fL]	0.15	0.07	−0.005	0.95
Fibrinogen [md/dL]	−0.11	0.23	0.08	0.39
PT [s]	0.32	<0.01	−0.05	0.55
INR	0.32	<0.01	−0.06	0.53
aPTT [s]	0.16	0.08	−0.08	0.38
D-dimers [mcg/mL]	0.31	<0.01	0.09	0.32
TT [s]	0.30	<0.01	−0.09	0.41
PLT [10^3^/µL]	−0.17	0.04	0.19	0.02

Values are Spearman’s coefficient of rank correlation (rho). aPTT—activated partial thromboplastin time, Hb—hemoglobin, Hct –hematocrit, ICU—intensive care unit, IQR—interquartile range, INR—international normalized ratio, MCH—mean cell hemoglobin, MCHC—mean cell hemoglobin concentration, MCV—mean cell volume, PLT—platelets, PT—prothrombin time, RDW—red cell distribution width, RDW-SD—red cell distribution width-standard deviation, SOFA—Sepsis-associated Failure Assessment score, TT—thrombin time.

## Data Availability

Data supporting reported results can be obtained from the corresponding author.
